# A 78‐year‐old woman with diffuse white matter infiltration and predominant involvement of bilateral temporo‐parieto‐occipital regions

**DOI:** 10.1111/jcmm.18245

**Published:** 2024-04-13

**Authors:** Charlotte Paoli, Anne Mc Leer, Julien Boyer, Lydiane Mondot, Bérengère Dadone‐Montaudié, Catherine Godfraind, Fanny Burel‐Vandenbos

**Affiliations:** ^1^ Laboratoire Central d'Anatomie Pathologique, Department of Pathology and Molecular Oncology University Hospital of Nice, Université Côte d'Azur Nice France; ^2^ Grenoble Alpes University Hospital Pathology Department Institute for Advanced Biosciences UGA/INSERM U1209/CNRS 5309, Grenoble Alpes University Grenoble France; ^3^ Laboratoire d'Anatomie Pathologique Centre de lutte contre le cancer Antoine Lacassagne Nice France; ^4^ Service d'imagerie médicale University Hospital of Nice, Université Côte d'Azur Nice France; ^5^ Laboratoire d'Oncologie moléculaire, Department of Pathology and Molecular Oncology University hospital of Nice, Université Côte d'Azur Nice France; ^6^ Neuropathology Unit University Hospital of Clermont‐Ferrand and University Clermont‐Auvergne, M2iSH UMR1071 Clermont‐Ferrand France

**Keywords:** DNA methylation, elderly patient, FGFR1 tandem duplication, paediatric‐type glioma

## Abstract

Diffuse paediatric‐type high‐grade glioma, H3‐wildtype and IDH‐wildtype (H3/IDH‐wt‐pHGG) is a newly defined entity amongst brain tumours, primarily reported in children. It is a rare, ill‐defined type of tumour and the only method to diagnose it is DNA methylation profiling. The case we report here carries new knowledge about this tumour which may, in fact, occur in elderly patients, be devoid of evocative genomic abnormalities reported in children and harbour a misleading mutation.

## 1 CLINICAL HISTORY

**FIGURE 1:**
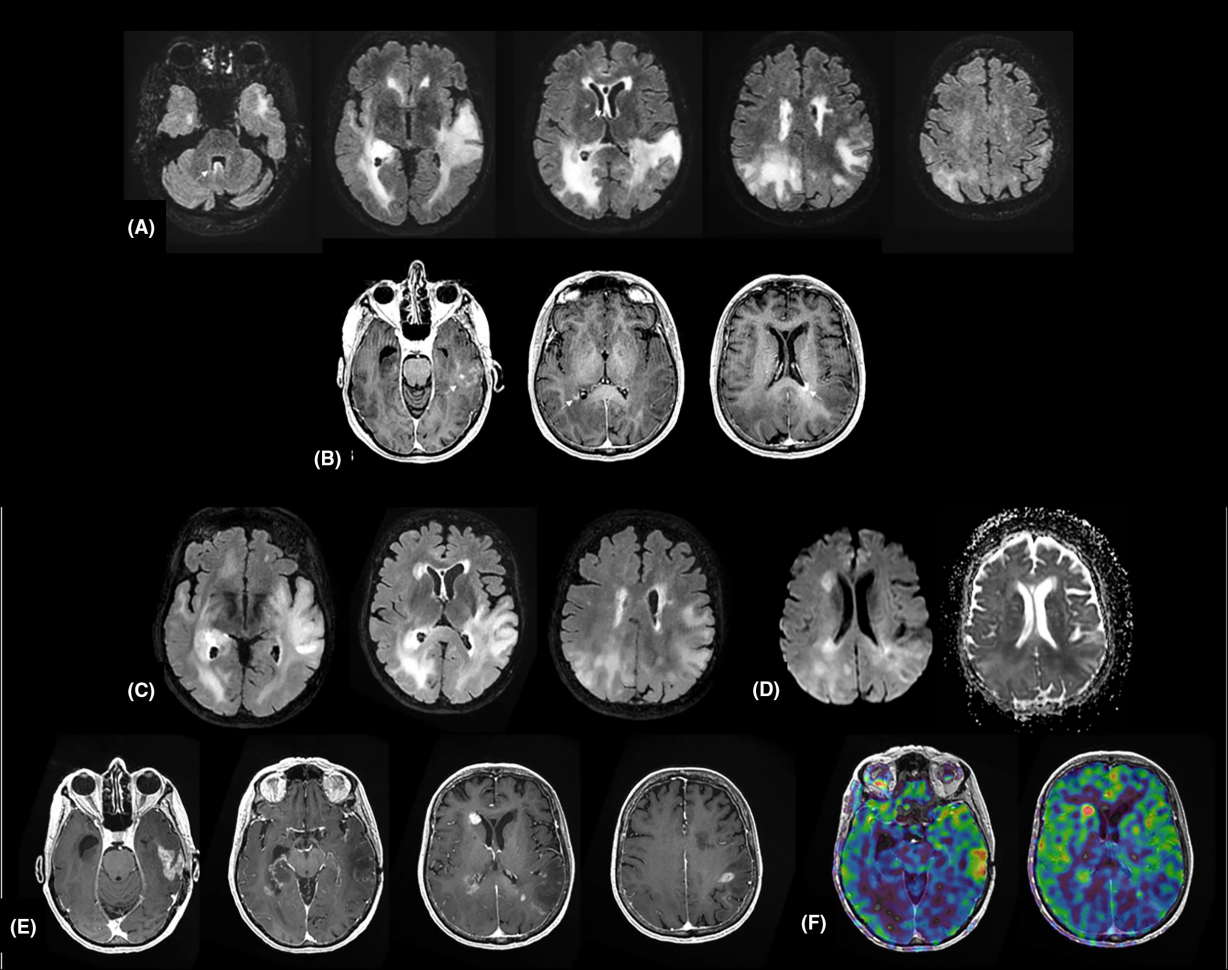
Radiological features. Preoperative brain MRI: (A) 3D FLAIR axial sequence – Diffuse white matter infiltration with predominant involvement of bilateral temporo‐parieto‐occipital regions, with cortical and ependymal extension (arrow). (B) Axial 3D T1‐weighted post‐contrast sequence: Foci of patchy enhancement, cortico‐subcortical left temporal and periventricular (arrow). Follow‐up imaging at 3 months postoperative under Temozolomide treatment: Increased tumour infiltration in T2 FLAIR imaging (C) with hypercellular foci showing hyperintensity on diffusion‐weighted imaging with decreased apparent diffusion coefficient (ADC) on mapping (D). Increased and appearance of enhancing foci (E), some of which are hypervascular, on ASL perfusion imaging (F).

A 78‐year‐old woman was admitted to the hospital with the chief complaint of language and behaviour issues for 3 weeks. Brain MRI showed a diffuse infiltration with predominant involvement of bilateral temporo‐parieto‐occipital regions in T2‐weighted sequence and foci of patchy enhancement after gadolinium injection (Figure 1A,B). Stereotactic biopsies were performed 10 days after the admission. The patient underwent treatment with temozolomide alone. Three months later, the evolution of the tumour was characterized by an increase in volume and an occurrence of necrotic and contrast‐enhanced areas on MRI (Figure 1C–F), as well as a clinical aggravation rapidly leading to the death of the patient.

## 2 FINDINGS

**FIGURE 2:**
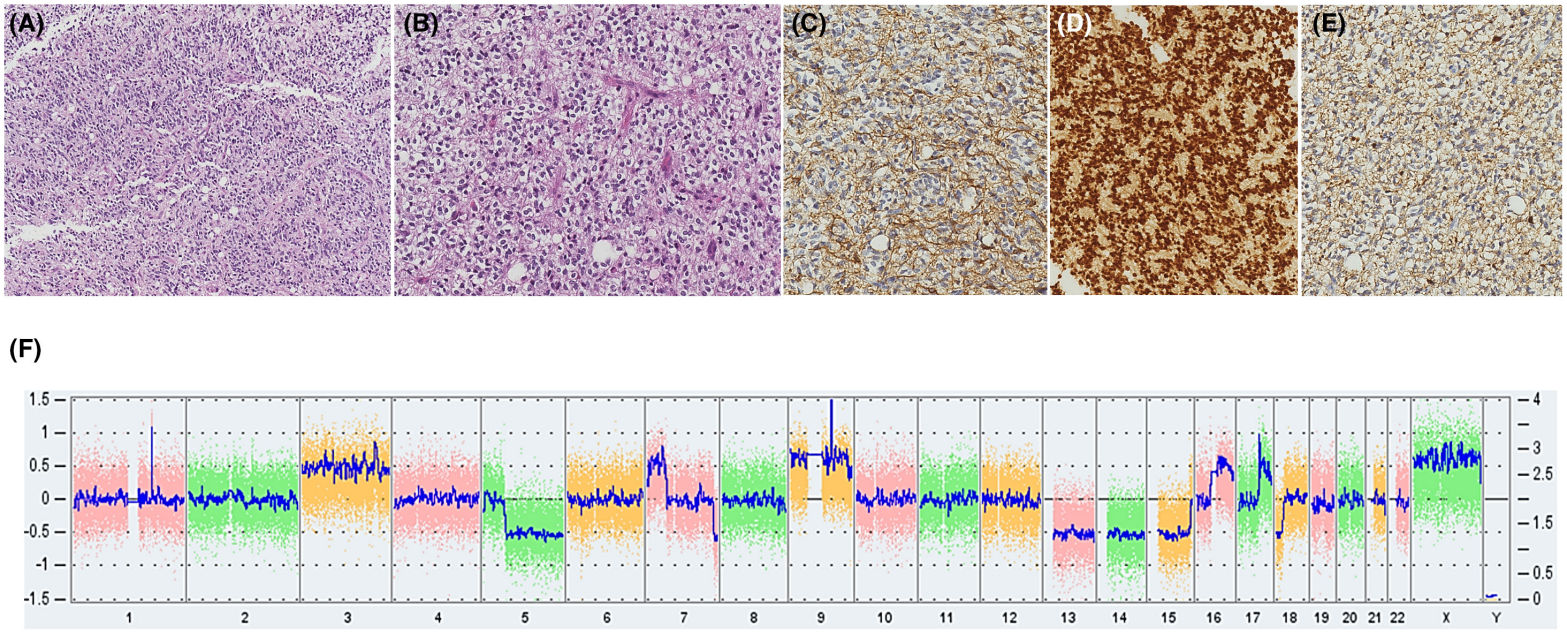
Histomolecular features. Histological and immunohistochemical features: (A) Diffuse glioma of moderate cellularity forming some perivascular pseudorosettes (×100). (B) Monotonous oligo‐like population of tumour cells (×200), immunopositive for GFAP (C, ×200), olig2 (D, ×200) and weakly positive for synaptophysin (E, ×200). Quantitative genomic profile in CGH/SNParray (F): Gains of whole chromosomes 3 and 9, segmental gain of chromosomes 7p, 16q and 17q, losses of whole chromosomes 13, 14 and 15, segmental losses of chromosomes 5q and 18p, amplification at 1q25 and 9q22.

Histological examination showed features of a moderately cellular tumour with some perivascular pseudorosettes, spongioblastic patterns and some microcalcifications without microvascular proliferation and necrosis (Figure 2A,B). It was composed of monotonous cells with delicate processes and an oligo‐like appearance. Vessels were small‐branched capillaries. After immunohistochemistry, tumour cells were positive for glial markers GFAP and Olig2 but negative for CD34, p53, synaptophysin, IDH1 R132H and FGFR3 (Figure 2C–E). ATRX was maintained. Proliferation index Ki67 was less than 1%. NGS and CGH/SNP array did not find any contributive molecular anomaly. Notably, there was no gain of Chromosome 7, no loss of Chromosome 10, no mutation was found in *TERT* promoter, *IDH1/IDH2*, *BRAF*, *H3F3A* and *HIST1H3B* genes (Figure 2F). In addition, no fusion was detected by RNA‐Seq (ArcherDx FusionPlex panel). Only a tandem duplication of *FGFR1* was detected by droplet PCR. By DNA methylation profiling, the methylation class in the brain classifier v12.8 of DKFZ was ‘Diffuse paediatric type high‐grade glioma, H3 wildtype and IDH wildtype’, ‘RTK1 subtype’ with scores at 0.90 and 0.83, respectively.

## 3 DIAGNOSIS

Diffuse paediatric‐type high‐grade glioma, H3 wildtype and IDH wildtype, World Health Organization (WHO) grade 4.

## 4 DISCUSSION

Diffuse paediatric‐type high‐grade glioma, H3‐wildtype and IDH‐wildtype (H3/IDH‐wt‐pHGG) has recently been introduced in the 2021 WHO Classification of Tumours of The Central Nervous System. This entity is a diffuse glioma with malignant features, mostly affecting children and adolescents. It is wildtype for the histone H3 genes as well as for *IDH1* and *IDH2*.^1^ DNA methylation profiling of this group defines three distinct subtypes termed pHGG RTK1, pHGG RTK2 and pHGG MYCN, which are enriched in *PDGFRA* amplifications, *EGFR* amplification/*TERT* promoter mutations and *MYCN* amplifications, respectively, whereas the chromosomal pattern typical of adult‐type glioblastoma Chromosome 7 gain (+7)/Chromosome 10 loss (−10)/EGFRvIII variant is absent. So far, DNA methylation profiling is the only method able to diagnose this new entity. As a rare and recently recognized tumour, H3/IDH‐wt‐pHGG remains to be further characterized in a clinicopathological point of view. Notably, its occurrence in the adult population remains unknown, as well as its imaging characteristics. Until the characterization by DNA methylation, the tumour in our case remained difficult to classify and the eventuality of a low‐grade glioma was not ruled out. Furthermore, the detection of a tandem duplication of *FGFR1*, an abnormality frequently associated with low‐grade gliomas, rendered the classification more confusing in this glioma of low‐grade morphology. Despite a high score, the result of DNA methylation profiling was initially doubtful due to discordance with the low‐grade morphology of the tumour and the context. However, this result ended up being consistent with the evolution of the tumour 3 months later evocative of an HGG. This observation is informative on an epidemiological and clinicopathological point of view. It shows that H3/IDH‐wt‐pHGG may occur, and are probably underestimated, in elderly patients, as recently observed by Bender et al.^2^ Moreover, H3/IDH‐wt‐pHGG may present as a diffuse glioma without frank enhancement on MRI. The bilateral involvement, present in our case, and described in children,^3^ could be a common presentation. Lastly, H3/IDH‐wt‐pHGG may present as a low‐grade glioma upon histological examination and may be associated with a potentially misleading *FGFR1* tandem duplication rather than the more evocative anomalies reported in this entity.

## AUTHOR CONTRIBUTIONS

**Charlotte Paoli:** Writing – original draft (equal). **Anne Mc Leer:** Formal analysis (equal); writing – review and editing (equal). **Julien Boyer:** Data curation (equal); formal analysis (equal); writing – review and editing (equal). **Lydiane Mondot:** Data curation (equal); formal analysis (equal); writing – review and editing (equal). **Bérengère Dadone‐Montaudié:** Data curation (equal); formal analysis (equal); writing – review and editing (equal). **Catherine Godfraind:** Data curation (equal); formal analysis (equal); writing – review and editing (equal). **Fanny Burel‐Vandenbos:** Conceptualization (lead); supervision (lead); writing – review and editing (lead).

## CONFLICT OF INTEREST STATEMENT

The authors declare no conflict of interest.

## PATIENT CONSENT

Each patient whose case is reviewed in the national Network RENOCLIP‐LOC is informed and gives consent.

## Data Availability

The data that support the findings of this study are available from the corresponding author upon reasonable request.
